# Essential Surgery at the District Hospital: A Retrospective Descriptive Analysis in Three African Countries

**DOI:** 10.1371/journal.pmed.1000243

**Published:** 2010-03-09

**Authors:** Moses Galukande, Johan von Schreeb, Andreas Wladis, Naboth Mbembati, Helder de Miranda, Margaret E. Kruk, Sam Luboga, Alphonsus Matovu, Colin McCord, S. Khady Ndao-Brumblay, Doruk Ozgediz, Peter C. Rockers, Ana Romàn Quiñones, Fernando Vaz, Haile T. Debas, Sarah B. Macfarlane

**Affiliations:** 1 Department of Surgery, College of Health Sciences, Makerere University, Kampala, Uganda; 2 Division of International Health (IHCAR), Karolinska Institute, Stockholm, Sweden; 3 Department of Surgery, Söder Hospital, Karolinska Institute, Stockholm, Sweden; 4 Department of Surgery, Muhimbili University of Health and Allied Sciences, Dar es Salaam, Tanzania; 5 School of Medicine, Catholic University of Mozambique, Beira, Mozambique; 6 Department of Health Management and Policy, University of Michigan School of Public Health, Ann Arbor, Michigan, United States of America; 7 Department of Anatomy, College of Health Sciences, Makerere University, Kampala, Uganda; 8 Kamuli Mission Hospital, Kamuli, Uganda; 9 Department of Surgery, Columbia University Medical Center, Columbia University, New York, United States of America; 10 Division of Pediatric Surgery, Hospital for Sick Children, University of Toronto, Toronto, Ontario, Canada; 11 Department of Epidemiology, School of Public Health, University of Michigan, Ann Arbor, Michigan, United States of America; 12 Higher Institute of Health Sciences, Maputo, Mozambique; 13 University of California, San Francisco Global Health Sciences, San Francisco, California; University of Queensland, Australia

## Abstract

**Background:**

Surgical conditions contribute significantly to the disease burden in sub-Saharan Africa. Yet there is an apparent neglect of surgical care as a public health intervention to counter this burden. There is increasing enthusiasm to reverse this trend, by promoting essential surgical services at the district hospital, the first point of contact for critical conditions for rural populations. This study investigated the scope of surgery conducted at district hospitals in three sub-Saharan African countries.

**Methods and Findings:**

In a retrospective descriptive study, field data were collected from eight district hospitals in Uganda, Tanzania, and Mozambique using a standardized form and interviews with key informants. Overall, the scope of surgical procedures performed was narrow and included mainly essential and life-saving emergency procedures. Surgical output varied across hospitals from five to 45 major procedures/10,000 people. Obstetric operations were most common and included cesarean sections and uterine evacuations. Hernia repair and wound care accounted for 65% of general surgical procedures. The number of beds in the studied hospitals ranged from 0.2 to 1.0 per 1,000 population.

**Conclusion:**

The findings of this study clearly indicate low levels of surgical care provision at the district level for the hospitals studied. The extent to which this translates into unmet need remains unknown although the very low proportions of live births in the catchment areas of these eight hospitals that are born by cesarean section suggest that there is a substantial unmet need for surgical services. The district hospital in the current health system in sub-Saharan Africa lends itself to feasible integration of essential surgery into the spectrum of comprehensive primary care services. It is therefore critical that the surgical capacity of the district hospital is significantly expanded; this will result in sustainable preventable morbidity and mortality.

*Please see later in the article for the Editors' Summary*

## Abstract 2

In the first of two papers investigating surgical provision in eight district hospitals in Saharan African countries, Margaret Kruk and colleagues find low levels of surgical care provision suggesting unmet need for surgical services.

## Editors' Summary

**Background:**

Every year, about 234 million major surgical procedures take place globally. Of these procedures, only a quarter are performed in low- and middle-income countries where nearly three-quarters of the world's population lives. Put another way, in high-income countries, 10,110 people out of every 100,000 have surgery each year on average compared to only 295 people out of every 100,000 in low- and middle-income countries. Yet conditions that need surgery (including complications of childbirth and traumatic injuries) are common in developing countries and contribute significantly to the burden of disease in these countries. Various organizations are working to reduce this burden by improving emergency and essential surgical care in developing countries. For example, the Bellagio Essential Surgery Group (BESG), which includes experts in surgery, anesthesia, obstetrics (the branch of medicine that cares for women during pregnancy and childbirth), and health policy from several African countries, the World Health Organization (WHO), and the US, aims to increase access to surgical services in sub-Saharan Africa.

**Why Was This Study Done?:**

One way to improve access to surgical services in sub-Saharan Africa would be to promote the provision of essential surgical services at district hospitals. These hospitals are the first referral facilities for people living in rural areas. Traditionally, patients receive much of their primary health care at these facilities but are referred to secondary and tertiary health care facilities (regional and national referral hospitals, respectively) for more specialized care. However, many surgical conditions—in particular, obstetric emergencies—need to be treated at district hospitals if lives are to be saved. Unfortunately, very little is known about the range and volume of surgical procedures currently undertaken in district hospitals in sub-Saharan Africa and such information is needed before programs can be developed to increase access to surgical services at these facilities. In this retrospective, descriptive study, the researchers (some of whom are part of the BESG) investigate the scope of surgery undertaken in district hospitals in three sub-Saharan African countries.

**What Did the Researchers Do and Find?:**

The researchers obtained recent data on the surgical procedures done at two representative district hospitals each in Tanzania and in Mozambique and four representative district hospitals in Uganda by examining hospital records and by interviewing administrators. The observed range of surgical procedures performed in these hospitals was narrow, they report, consisting mainly of essential and life-saving emergency procedures such as cesarean sections for the delivery of babies and wound-related procedures. Obstetric procedures accounted for around half of all surgical procedures in all the hospitals except one Ugandan hospital. Hernia repair and wound care accounted for nearly two-thirds of general surgical procedures. The surgical output across the hospitals varied from five to 45 major procedures per 10,000 people in the population (average 25 operations per 10,000 people). Across the hospitals, between one and 17 cesarean sections and between 0.5 and seven hernia repairs were performed per 10,000 people in the population. Finally, the researchers used their data and WHO estimates of the population need for cesarean sections to estimate that in the two Tanzanian district hospitals, between half and two-thirds of women that needed a cesarean section did not have access to this life-saving procedure.

**What Do These Findings Mean?:**

These findings suggest that there are low levels of provision of surgical care in district hospitals in Tanzania, Mozambique, and Uganda. Further studies are needed to confirm that these findings are generalizable to district hospitals elsewhere in sub-Saharan Africa and to quantify the extent to which this low level of surgical care translates into unmet needs. Limitations of the study include a lack of information on outcomes, on referral of patients to higher-level facilities, and on how many of the surgical procedures undertaken at these hospitals dealt with traumatic injuries. Nevertheless, the information collected in this study, together with that in a separate paper that investigates the availability of health workers and funding for the provision of essential surgery in district hospitals in these three countries, suggests that the surgical capacity of district hospitals in sub-Saharan Africa needs to be improved. If this goal can be achieved, suggest the researchers, it should avert many illnesses and deaths in this poor region of the world.

**Additional Information:**

Please access these Web sites via the online version of this summary at http://dx.doi.org/10.1371/journal.pmed.1000243.

- The availability of health workers and funding for surgery in the same hospitals is investigated in a *PLoS Medicine* Research Article by Margaret E. Kruk et al.
- Information on the Bellagio Essential Surgery Group is available
- WHO's Global initiative for Emergency and Essential Surgical Care plans to take essential emergency, basic surgery and anesthesia skills to health care staff in low- and middle-income countries around the world; WHO also has a page describing the importance of emergency and essential surgery in primary health care

## Introduction

The current drive towards a health systems approach for delivering health care interventions in Africa opens an opportunity to redress long-standing neglect in the provision of surgical services [1],[2]. Health systems must be flexible enough not only to prevent and treat the high morbidity and mortality from infectious diseases and the increasing prevalence of noncommunicable conditions, but also to alleviate conditions requiring prompt surgical interventions such as obstetric emergencies and trauma cases [3]. The traditional referral-based system in which patients are provided primary health care at first-level referral facilities and referred to secondary and tertiary health care facilities for specialized care has undermined the capacity to provide timely access to essential surgical procedures. Although some surgical conditions can be postponed until the patient can gain access to specialized care, others result in death or severe disability if treatment is delayed. This situation is clearly highlighted in the case of obstetric emergencies in which it is recognized that in order to prevent maternal deaths, cesarean sections can be provided at some first referral facilities, and must be provided at district hospitals. We argue that the same basic surgical skills and equipment needed to perform emergency obstetric procedures are required to treat other surgical conditions and therefore that building surgical capacity has the potential to improve a range of health outcomes.

As funders and national policymakers consider the expansion of health systems in sub-Saharan Africa, accurate data on the current scope of surgery at district hospitals or first-level referral facilities are needed to inform programs to increase access. However, such data are limited or missing altogether. In particular, although the district hospital is the designated first-level provider of surgical services in many African countries, there is only partial information on the nature and volume of surgery performed [4]. There are no population-based studies in the sub-Saharan context that estimate the need for surgical procedures.

This article aims to contribute to the nascent literature on the role of the district hospital in providing essential surgery by describing the scope of surgery at district hospitals from three countries in eastern Africa.

## Methods

### Selection Criteria

We conducted a retrospective descriptive survey of eight hospitals in three African countries represented at the inaugural meeting of the Bellagio Essential Surgery Group (BESG) in Bellagio, Italy in 2007 [2]: Tanzania, Uganda, and Mozambique (see map in Figure S1). We chose at least two hospitals in three countries to assess variability in service provision. The hospitals were required to be designated district hospitals funded and operated by the government, have 50–150 beds, serve a rural catchment population, and in existence for at least 2 y. Finally, the hospitals had to have cost and utilization data available for 2006 or 2007.

### Study Sites

Tanzania, Uganda, and Mozambique are of comparable size and per capita income, and have similar basic public health indicators (Table 1). The health service delivery system in these countries is regionalized and structured as a pyramid with the dispensary at the lowest level and the national referral hospital at the highest level. Health centers, district hospitals, and regional hospitals serve as intermediaries between levels.

**Table 1:** Demographic profile of three African countries under investigation, 2008 [25],[26].

Country	Uganda	Mozambique	Tanzania
**Population (millions)**	31.4	21.3	40.2
**Growth rate (%)**	3.6	1.8	2.1
**GNP per capita (US$)**	276	310	350
**Birth rate** a	48	38	35
**Literacy rate (%)**	66	48	69
**Death rate** b	12.3	20.3	12.9
**Infant mortality rate** c	66	108	70
**Life expectancy (y)**	52	41	51
**Fertility rate** d	6.8	5.2	4.6
**Maternal mortality rate** e	550	950	520

a Birth rate is the ratio of total live births to total population in a specified community or area over a specified period of time. The birth rate is often expressed as the number of live births per 1,000 of the population per year.

b Death rate is the ratio of total deaths to total population in a specified community or area over a specified period of time. The death rate is often expressed as the number of deaths per 1,000 of the population per year.

c Infant mortality rate is the death rate during the first year of life, the ratio of deaths in an area to the population of that area; expressed per 1,000 per year.

d Fertility rate is the average number of children that women have during their lives, from age 15 y to age 50 y.

e Maternal mortality rate is the number of pregnancy-related deaths/100,000 women of reproductive age; the number of maternal deaths related to childbearing divided by number of live births—or number of live births + fetal deaths per year.

In Tanzania we studied Bagamoyo and Kasulu District Hospitals. Bagamoyo is a district hospital about 60 km from the national referral hospital in Dar es Salaam. It serves a population of 230,000 people and has five satellite health centers. Kasulu, on the other hand, is situated about 1,500 km from Dar es Salaam, in the western Tanzania region of Kigoma. It serves a population of 600,000 people and has nine satellite health centers.

In Uganda, we studied Mityana, Kiryandongo, Iganga, and Buluba District Hospitals. Mityana, Iganga, and Kiryandongo hospitals are situated astride Ugandan highways, 72 km, 110 km, and 200 km, respectively, from the capital city of Kampala. They serve suburban and rural populations. The three hospitals are officially 100-bed hospitals with catchment populations of 250,000 (for Mityana), 125,000 (for Kiryandongo), and at least 500,000 (for Iganga). Buluba and Iganga serve the same region away from Kampala.

In Mozambique, we studied Chokwe and Catandica District Hospitals. Chokwe hospital is situated in the Gaza province 150 km northeast of the capital city of Maputo. The hospital, along with its nine satellite health centers, serves a district population of 250,000 people. Catandica is situated in the Manica province, approximately 1,000 km northwest of Maputo, abutting the mountainous border with Zimbabwe. Catandica hospital serves a catchment population of approximately 100,000 people and has ten satellite clinics. Although Chokwe is well connected to nearby towns and is located 90 km from its province capital city of Xai-Xai, Catandica is situated in a remote region off the unpaved main road, 200 km from its province capital of Chimoio. Both hospitals serve primarily rural populations.

### Data Collection

Data were collected from country-level censuses, administrator interviews, hospital records, and administrative records using a standard survey instrument, categorized as: district demographics, hospital characteristics including inputs (staffing, cost, functioning and nonfunctioning beds, operating rooms, etc.), and outputs (aggregate and individuals data on admissions and procedures). District population data were gathered from the most recent census in each country. The number of other hospitals and health centers in the district was obtained from local hospital administrators.

Comprehensive hospital-level information about human resources was collected: number of doctors, surgeons, dentists, mid-level health providers (MLPs) including technicians, assistant medical officers, clinical officers), nurses (including the subcategories of nurse officers, nurses' assistants, enrolled nurses, nurse midwives, public health nurses), as well as administrative and support personnel. Data on other hospital inputs (e.g., functioning and nonfunctioning beds, operating rooms, hospital operating expenditures for 2006 and/or 2007) also came from central administration records and administrator interviews in each hospital. Where 2007 data were not available (i.e., in Uganda), 2006 data were used.

Data on admissions and procedures were collected from two sources. First, we collected aggregate hospital statistics for 2007 on annual admissions, deliveries, and major surgeries from existing hospital information systems (which produce monthly statistics). Second, we collected patient-level information, including age and sex. For five hospitals the patient-level data were collected for the target months of February, June, and October, whereas for Mityana, Buluba, and Iganga hospitals, data were collected for a full 12 mo. We reviewed ward-level patient records from medical, surgical, maternity, and pediatric wards to determine patient diagnosis, age, and gender at admission. We also reviewed operating room logs for surgical procedure details. To standardize data entry and case definition, all sites used the same list of most common procedures and diagnoses on the basis of a consensus panel of physicians in the respective countries. A category “other” was only possible if none of the listed choices were appropriate. When “other” data were collected, an open-ended description was required. Ethical clearance was not required as all data were de-identified data previously collected for administrative purposes.

### Data Processing and Analysis

Aggregate hospital statistics on annual surgeries were used to describe the scope of surgical procedures. Ward-level patient records for the targeted months of February, June, and October were used to describe the age and gender distribution by type of procedure. We compared the percent distribution of each procedure obtained from the annual aggregate data to that of 3-mo individual ward-level data. Few discrepancies were observed for Mityana, Buluba, and Kiryandongo, where annual individual data were available. More information about specific discrepancies is presented in Table S1. All data were entered in Microsoft Excel at each of the sites and analyzed centrally using Excel and SPSS.

## Results

Catchment populations ranged from about 99,000 to 628,000 people (Table 2). Admissions ranged from 3,861 to 16,999 and deliveries from 439 to 3,607. Facilities were staffed with one to six doctors and three to 26 mid-level health providers. The number of hospital beds per 1,000 population ranged from 0.2 to 1.

**Table 2:** Characteristics, outputs, and human resources for eight district hospitals in Tanzania, Mozambique, and Uganda, 2008.

Country	District	Population^a^	Hospital Characteristics and Outputs							Human Resources			
			Beds	Bed/1,000	Admissions	Inpatient Days	Outpatient Visits	Normal Deliveries	Average Length of Stay	Doctors	MHLP		Nurses
											AMO^b^	CO^c^	
**Tanzania** a	Bagamoyo	230,164	125	0.5	6,545	23,332	39,441	1,763	3.56	5	8	11	51
	Kasulu	628,677	135	0.2	10,296	58,293	42,456	2,505	5.66	1	13	11	116
**Mozambique** d	Chokwe	226,049	214	1.0	8,089	28,764	44,018	2,518	3.56	4	1	3	31
	Catandica	99,546	91	0.9	3,861	15,209	37,624	1,699	3.94	1	3	4	18
**Uganda** a	Mityana	269,763	100	0.4	9,106	38,245	41,496	3,108	4.2	6	9	0	73
	Kiryandongo	125,000	100	0.8	5,713	24,566	44,546	1,037	4.3	2	3	0	75
	Buluba	388,052	120	0.3	6,310	36,446	37,248	439	5.8	4	0	3	24
	Iganga	547,155	157	0.3	16,999	51,971	70,469	3,607	3.1	5	0	8	71

Data are based on annual aggregate hospital statistics extracted from hospital information systems.

a 2007 country-level census data.

b Assistant medical officers (AMOs), nonphysician medical professionals with at 5–6 y postsecondary education assuming many clinical responsibilities.

c Clinical officers (COs), nonphysician medical professionals with 3 y postsecondary education assuming roles similar to those of advanced nurses.

d 2002 country-level census data.

MHLP, mid-level health provider.

The annual aggregate number of surgical procedures performed per hospital is given in Table 3. With the exception of Kiryandongo, obstetric and nonobstetric procedures accounted for approximately 40%–60% of all procedures. In six of the eight hospitals, more than 60% of nonobstetrical procedures were major procedures.

**Table 3:** Breakdown of the annual number of surgeries performed at hospitals under investigation.

Country, Region, and Year	Nonobstetric Operations		Obstetric Operations			Total Operations	Major Procedures/10,000
	Total (% of All Procedures)	Major Operations (% of Nonobstetric Procedures)	Total (% of All Procedures)				
**Tanzania 2007**							
Bagamoyo	549 (66)	428 (78)	431 (44)			980	38
Kasulu	1,162 (57)	242 (21)	883 (43)			2,045	18
**Mozambique 2007**							
Chokwe	224 (37)	171 (76)	377 (63)			601	25
Catandica	146 (57)	133 (91)	110 (43)			256	24
**Uganda 2006**							
Mityana	730 (49)	456 (63)	754 (51)			1,484	45
Kiryandongo	213 (86)	80 (38)	35 (14)			248	5
Buluba	139 (58)	125 (90)	100 (42)			239	6
Iganga	920 (50)	711 (77)	915 (50)			1,835	30

Data are based on annual aggregate hospital statistics extracted from hospital information systems.

**Figure 1:**
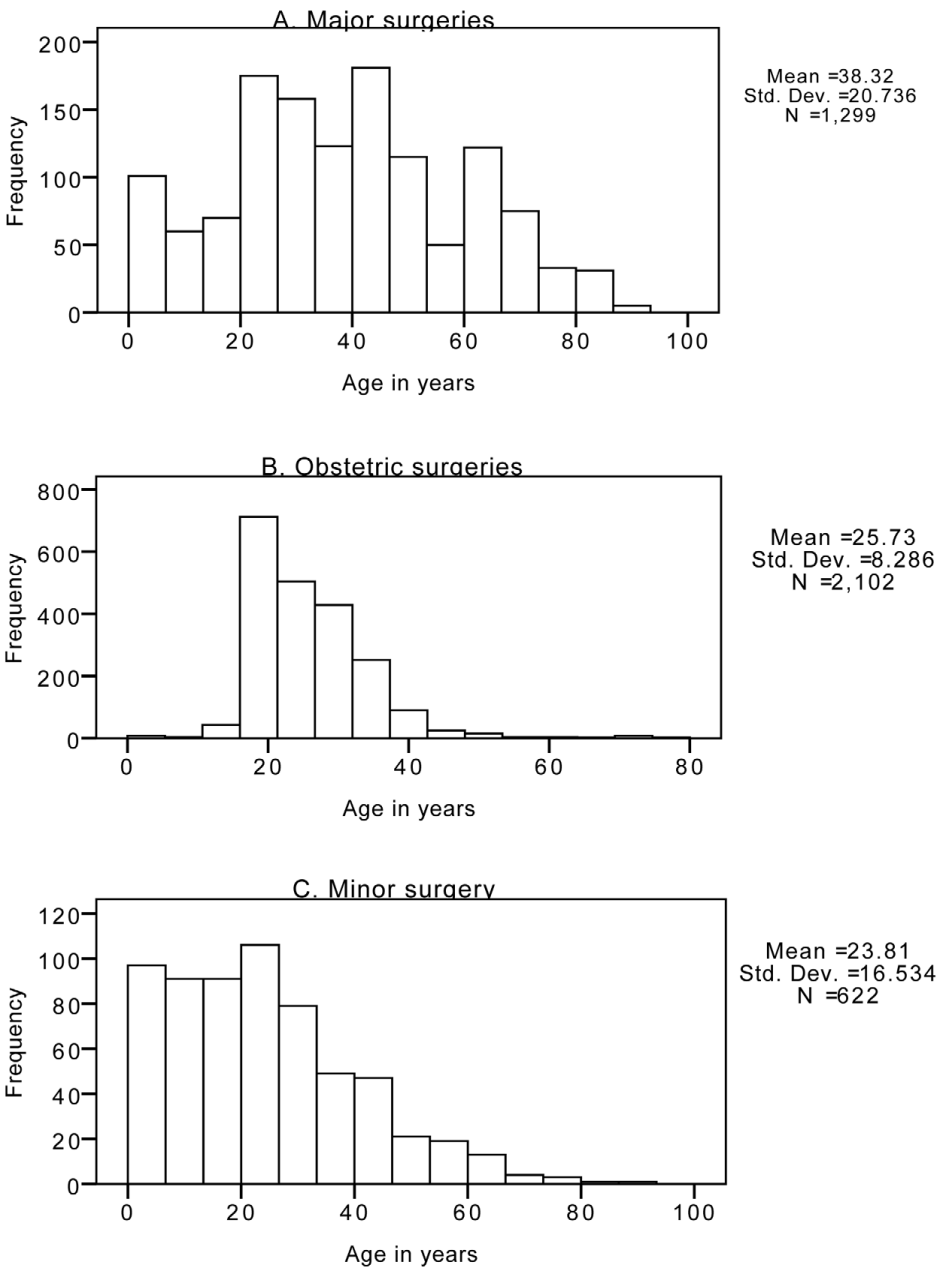
Age distribution of procedures by type of surgery across all hospitals under investigation. (A) Major surgeries; (B) obstetric surgeries; (C) minor surgery. Data are extracted from ward-level patient records for the targeted months of February, June, and October.

**Figure 2:**
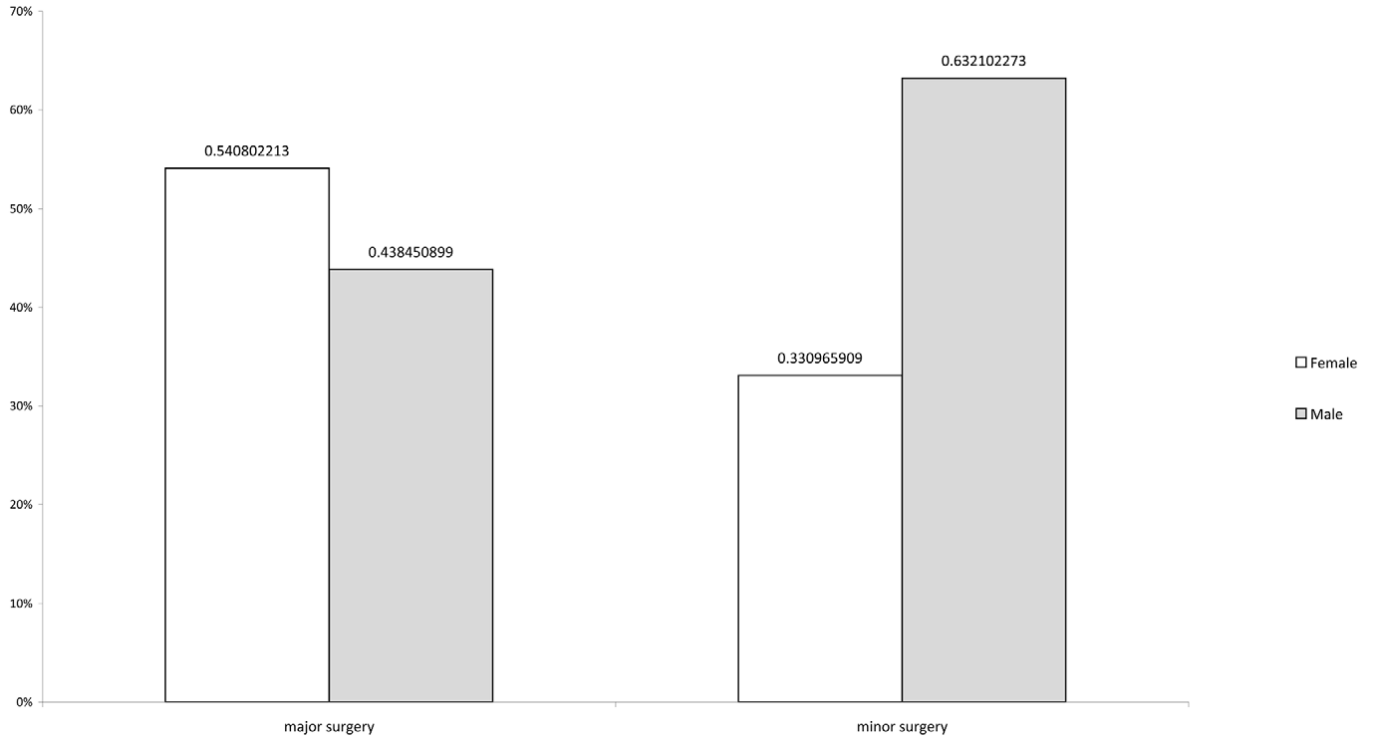
Type of surgery by patient sex. Data are extracted from ward-level patient records for the targeted months of February, June, and October; 100% of obstetric surgeries were performed in female patients; 71.9% of unspecified surgeries were in female patients versus 23.1% in males. The numbers do not add up to 100% because of missing data on sex of the patient.

The total range of procedures when summed across all hospitals is displayed in Tables 4 and 5. Figure 1 shows the age distribution for persons receiving major, minor, and obstetric operations across all sites. Major surgery was performed across all age groups but occurred more frequently among patients aged 25 to 50 y with an average age of 40 y. Minor surgery was more common for patients under 50 y old with an average age of 24 y. The average age of patients on whom obstetric operations were performed was 26 y. Figures 1 and 2 show the age distribution for patients receiving major operations at each site, and the gender distribution for both major and minor surgeries. The differences in average ages reflect, in part, the differences in the proportions of procedures performed at each site (see Table S1).

**Table 4:** Breakdown of major surgical procedures at hospitals under investigation.

Procedure	Tanzania 2007		Mozambique 2007		Uganda (2006)			
	Bagamoyo	Kasulu	Chokwe	Catandica	Mityana	Kiryandongo	Buluba	Iganga
**Major nonobstetric procedures (** ***n*** **)**	(428)	(242)	(171)	(133)	(456)	(80)	(125)	(711)
**Amputation**	—	3%	2%	8%	—	1%	10%	1%
**Appendectomy**	11%	2%	6%	2%	2%	—	1%	4%
**Circumcision**	—	1%	13%	18%	1%	68%	1%	—
**Excision**	—	10%	—	5%	—	—	—	—
**Herniorrhaphy (all)**	22%	24%	17%	20%	41%	16%	24%	29%
**Hydrocelectomy**	13%	8%	4%	20%	2%	1%	4%	0%
**Hysterectomy (gynecologic diagnoses)**	6%	2%	9%	—	5%	1%	2%	17%
**Laparotomy (all)**	6%	26%	20%	10%	3%	5%	10%	43%
**Open fracture reduction**	3%	—	2%	—	31%	—	—	—
**Other**	9%	24%	29%	17%	15%	8%	48%	5%
**Obstetric procedures (** ***n*** **)**	(431)	(883)	(377)	(110)	(754)	(35)	(100)	(915)
**Bilateral tubal ligation**	6%	11%	7%	4%	10%	14%	—	—
**Cesarean section**	61%	62%	80%	73%	63%	74%	88%	88%
**Evacuation** a	30%	22%	—	1%	19%	—	—	0
**Other**	3%	5%	13%	23%	8%	11%	12%	12%

Data are based on annual aggregate hospital statistics extracted from hospital information systems.

a Some evacuations may not have been recorded in the operating room logbooks (the source of data for this study) as they are not always carried out in the main operating rooms.

**Table 5:** Range of all procedures performed across all hospitals under investigation.

Procedures	Number of Procedures	Range (%) of All Procedures at a Hospital
**Cesarean section**	2,583	10–50
**Wound-related procedures** a	1,675	10–50
**Herniorrhaphy**	642	5–13
**Uterine evacuation and D&Cs**	556	0–13
**Laparotomy**	475	1–17
**Open fracture reduction**	285	0–15
**Bilateral tubal ligation**	237	0–5
**Hysterectomy (gynecologic diagnoses)**	193	0–7
**Other major surgeries**	174	0–17
**Hydrocelectomy**	138	0–11
**Circumcision**	110	0–22
**Appendectomy**	101	0–5
**Excisional biopsies of superficial lumps**	74	0–4
**Salpingectomy**	70	0–3
**Other obstetric surgeries**	67	0–3
**Norplant removal**	52	0–4
**Limb amputation**	40	0–5
**Skin graft**	40	0–3
**Extraction of placenta**	33	0–1
**Cystostomy**	23	0–1
**Emergency obstetric hysterectomy**	21	0–2
**Osteotomy/sequestrectomy**	19	0–5
**Repair of ruptured uterus**	16	0–4
**Foreign body removal**	13	0–2
**Other minor surgeries**	10	0–1
**Orchiectomy**	9	0–0.5
**Haemorrhoidectomy**	8	0–0.4
**Splenectomy**	6	0–0.1
**Lumpectomy & mastectomy**	6	0–0.1
**Open prostatectomy**	3	0–0.8
**Cervical tear repair**	2	0–0.1
**Vesico-vaginal fistula repair**	2	0–0.4
**Others**	2	0–0.4

Data are based on annual aggregate hospital statistics extracted from hospital information systems.

a Wound-related procedures include those recorded as surgical toilet and suture (washout and closure of a wound); incision and drainage (often an abscess); wound dressing; and wound debridement.

D&C, dilation and curettage.

The median annual rate of major operations across the eight hospitals was 25/10,000 population (range 5–45) (Table 3). Table 6 shows that the range of cesarean sections per 1,000 deliveries in these hospitals was 25–223, whereas the range of cesarean sections/10,000 population was 1–17. Meanwhile, 0.5–7 hernia repairs were performed across the hospitals per 10,000 population. The population need for cesarean sections is debatable, but is estimated by the WHO to be 5%–15% of all deliveries [2]. Using a rate of 5% and a birth rate of 35/1,000 (estimated for Tanzania in Table 1), we calculated the annual need of cesarean sections for the catchment populations (given in Table 2) of Bagamoyo and Kasulu to be 400 and 1,100 cesarean sections, respectively. These calculations imply that the unmet need for cesarean section in Bagomoyo was 65%, whereas for Kasulu it was 47%.

**Table 6:** Annual rates of hernia operations and cesarean operations/10,000 population.

Country and Year	District Hospital	Herniorrhaphy		Cesarean Section		
		Frequency	Operations/10,000 Population	Frequency	Operations/10,000 Population	Operations/1,000 Deliveries
**Tanzania 2007**	Bagamoyo	94	4.8	263	11.43	149
	Kasulu	58	0.92	545	8.67	218
**Mozambique 2007**	Chokwe	29	1.28	301	13.32	119
	Catandica	26	2.61	80	8.04	47
**Uganda 2006**	Mityana	188	6.97	475	17.61	153
	Kiryandongo	13	0.52	26	1.04	25
	Buluba	30	0.77	88	2.27	200
	Iganga	204	3.73	805	14.71	223

Data are based on annual aggregate hospital statistics extracted from hospital information systems.

## Discussion

We found relatively low rates of major surgery at district hospitals in East Africa, ranging from 50 to 450 surgical procedures per 100,000 population. Although lack of data on the population need for surgery precludes conclusions about the magnitude of unmet need, this confirms that there are barriers to access to essential surgery in at least some rural districts in Uganda, Tanzania, and Mozambique. This is reinforced by our finding that the majority of nonobstetric surgery was for emergency rather than elective conditions, suggesting that district residents do not receive surgical care for common surgical conditions (e.g., inguinal hernia repair) in local hospitals.

Global data also point to limited access to essential surgery in most low-income countries, with only 26% of 234 million estimated surgical procedures performed in poor and lower health expenditure countries that account for 70% of the world's population [4]. Nonetheless, very little is known about the incidence and prevalence of surgical conditions, even the number of basic surgical conditions such as hernia. Prior facility-based reports of surgical procedures in low-income countries have focused on individual institutions or countries, but there have not been cross-country comparisons using a common template [4]–[9]. In many of the sites investigated the number of public hospital district beds have remained the same for decades, while the population on average has doubled over the period [10]. This has meant, in effect, a decline in hospital beds per population over time.

At the first meeting of the BESG, participants drafted a list of the highest-priority surgical procedures that could be carried out at the level of the district hospital (first-level health facility) in Africa [11]. These procedures heavily overlap with the set of procedures defined by the World Health Organization [2]. These highest priority procedures primarily address health emergencies such as injuries and obstetric complications. A recent estimate suggests that injuries account for the greatest mortality and morbidity from surgical conditions in Africa (15 disability-adjusted life years [DALYs] lost per 1,000 population) [12]. At the same time prior work in similar settings has suggested that only one-third of injured patients reach a health facility [12].

Our data suggest but do not definitively confirm that the volume of surgical procedures to treat traumatic injury was relatively low in the study hospitals. For example, while some of the laparotomies performed were likely due to trauma, they were not clearly classified as such, and may have been performed for other causes of acute abdomen such as bowel obstruction or intestinal perforation. Open fracture reduction, which might also be considered a proxy for trauma when treated at a district hospital, was recorded at only two of the eight hospitals (Bagamoyo and Mityana). Similarly, there were no burns recorded and very few skin grafts, though burns in rural areas are a well-documented serious public health issue, especially in children [13]. Life-saving interventions such as airway management, tube thoracostomy for chest trauma, and resuscitation with intravenous fluids were not recorded, as the data do not include bedside and some accident and emergency procedures. The difficulties we encountered in obtaining data on trauma suggest the need for new data collection approaches. Trauma registries have been piloted in several countries in the region and other groups have called for greater facility-based and population-level surveillance of injuries to improve knowledge of trauma care [14]–[16]. The few existing population-based surveys of injuries have pointed to a high incidence of untreated injuries [12],[17].

In terms of obstetric surgery it is notable that cesarean section births, while still insufficient to meet the population need, far outnumbered instrumental deliveries (e.g., vacuum extraction), which were extremely rare in the study hospitals. This finding raises a concern for the quality of obstetric care as instrumental delivery poses lower risk to the mother and the fetus, particularly in the context of weak infection control practices and high rates of repeat childbearing for women [18]. There also appeared to be a gap in the provision of surgical family planning. No vasectomies were recorded across the study hospitals. The tubal ligation rate in the hospitals translates to 90 per million compared to 1,150 per million in the US [19]MLHP By reducing the rate of unwanted pregnancy, surgical and nonsurgical family planning methods can reduce the need for surgical interventions for birth complications [20]. Similarly very few cancer-related procedures were done in the study hospitals. It is unclear from these data if these limitations are due to a lack of capacity at the district hospital, to referral to higher-level facilities, or to low health-seeking behavior. There were no accessible records to establish what kinds of conditions were referred to higher-level hospitals over the study period. The same is true for other elective conditions known to be prevalent in the region such as vesico-vaginal fistula [21]. Another possibility discussed under limitations is that the data for these procedures were not captured in the operating room logs.

In seven of the eight studied hospitals the surgeries performed were evenly divided between obstetric and nonobstetric procedures. This finding is in contrast to a recent survey of 21 district hospitals in Malawi that found considerably more obstetric procedures followed by surgical toilet and suture and fracture manipulation [9]. In our study, wound-related procedures were the second most commonly performed operation after cesarean sections, followed by herniorrhaphies. While the ratio of cesarean sections to laparotomies was 13∶1 in the Malawi study, it was 5∶1 in our study. More laparotomies were performed in central hospitals in Malawi, perhaps reflecting different patterns of practice and referral.

All of the figures on surgical provision should be interpreted in the context of the population need for surgery. However, while it is possible to estimate unmet need for emergency obstetric care (see Results section), it remains difficult to estimate the expected need for general surgery in sub-Saharan Africa. For one, we have limited information on the population served by each hospital and there may be other facilities that provide services or patients may travel to other hospitals. In the absence of estimates of unmet need, we compared our findings with surgical rates per population obtained in other countries. We found that surgical rates/catchment population in this study (60–450/100,000) were comparable to prior estimates in the region (263/100,000 in Kenya; 166/100,000 in Uganda; 369/100,000 in Malawi) [4]–[9].

Kiryandongo hospital in northern Uganda was an outlier with very low surgical output. According to on-site interviews the hospital had deficits in staffing of essential cadres for surgical services such anesthetic and medical officers. In addition, the population in the catchment area includes several large camps of refuges from Southern Sudan and a native Ugandan population, some of whom seek care in another regional hospital where a different local dialect is spoken.

The age distribution from these data, especially for major procedures, indicates that the young productive workforce receives the majority of surgeries (Table 1). There are few procedures recorded in the pediatric age group. This finding may suggest a significant unmet need for pediatric surgery given that up to half the population is less than 15 y of age in many of these countries, and that children are more prone to injuries and congenital anomalies. One study estimated that up to 85% of children living in an urban areas of the Gambia are likely to need a surgical procedure by age 15 y, generally minor surgery [3].

Most of the recorded procedures were for emergency surgical conditions, suggesting significant potential mortality and morbidity averted through the provision of surgical care. However, this finding leads to the question of why numbers of elective procedures were low. The one exception is hernia surgery, which was relatively common. Again, it is not known how many of the herniorrhaphies performed were for strangulated hernia, an emergency condition, as this was not recorded in all hospitals. Whether patients with less acute surgical conditions do not seek care at all or are referred to higher-level facilities requires further investigation.

The limitations of this study include the usual limitations of using retrospective hospital records, for example the low number of minor operations observed may be because they were recorded as outpatient procedures not in the theatre logbooks. This practice may account for the absence of evacuations of remnants of pregnancy in the records as these may have been treated outside the theatre. In addition our study lacked information on outcomes of operations. The limited evidence available suggests that perioperative morbidity and mortality from procedures are significant in these settings and makes the case that anesthesia services and safety measures must also be stressed [22]–[24]. In addition, accurate estimates of patient referrals to higher-level facilities for conditions that exceeded the capacity of the district hospitals could not be assessed through this study. This absence may result in overestimates of unmet need as these estimates were made assuming that all cesarean sections required in the district population were done at the index hospitals. The estimate of unmet need for cesarean sections may have been inflated by some operations that were performed for convenience rather than need. Also, there may have been misclassification of operations as there is no standardized way that operations are recorded. For example, “surgical toilet” could mean a major complicated dirty wound or a simple laceration. This misclassification could be addressed by future prospective facility-based data collection. Finally, the age and gender distribution of the surgeries should be interpreted in light of the discrepancies observed between the 12-mo aggregate data and the 3-mo individual data used to estimate patient demographics.

Although the scope of surgical procedures done in these first-level referral facilities in Tanzania, Mozambique, and Uganda is narrow, emergency and essential procedures were the primary procedures performed. This study did not formally assess deficiencies in infrastructure and equipment, but qualitative interviews and the experience of the authors confirm that these procedures are performed under very challenging circumstances. Many procedures designated as high priority by the World Health Organization and other groups are performed in low volume or not at all. In particular, there is a low volume of essential trauma care such as fracture management, and of management of procedures for cancer, and for vesico-vaginal fistula repair.

This study suggests the need for urgent new research into the provision of essential surgery at the district level. Our results are limited as they are based on retrospectively collected data but are useful in pointing to the need for carefully designed prospective studies. The unmet need for surgical care could be estimated by population-based surveys and measurement of surgical need should be included in such surveys. Outcomes of care and referral patterns could be better assessed by prospective studies of first- and referral-level facilities. The district hospital is well positioned in the health service delivery hierarchy to integrate surgery and primary health care. The surgical capacity of the district hospital therefore must be strengthened to avert morbidity and mortality from surgical conditions. Greater emphasis must be paid to the role of surgical care in the global health research agenda and in plans to improve health care for vulnerable rural populations in sub-Saharan Africa.

## Supporting Information

Figure S1 Map of locations of hospitals under investigation.
(0.97 MB TIF)

Table S1 Discrepancies between the 3-month individual and 12-month aggregate data.
(0.05 MB DOC)
