# Supplementary figures and images for: Essential Surgery at the District Hospital: A Retrospective Descriptive Analysis in Three African Countries
Source: PLoS Med. 2010 Mar 9;7(3):e1000243. doi: 10.1371/journal.pmed.1000243 (PMC2834708; doi:10.1371/journal.pmed.1000243)

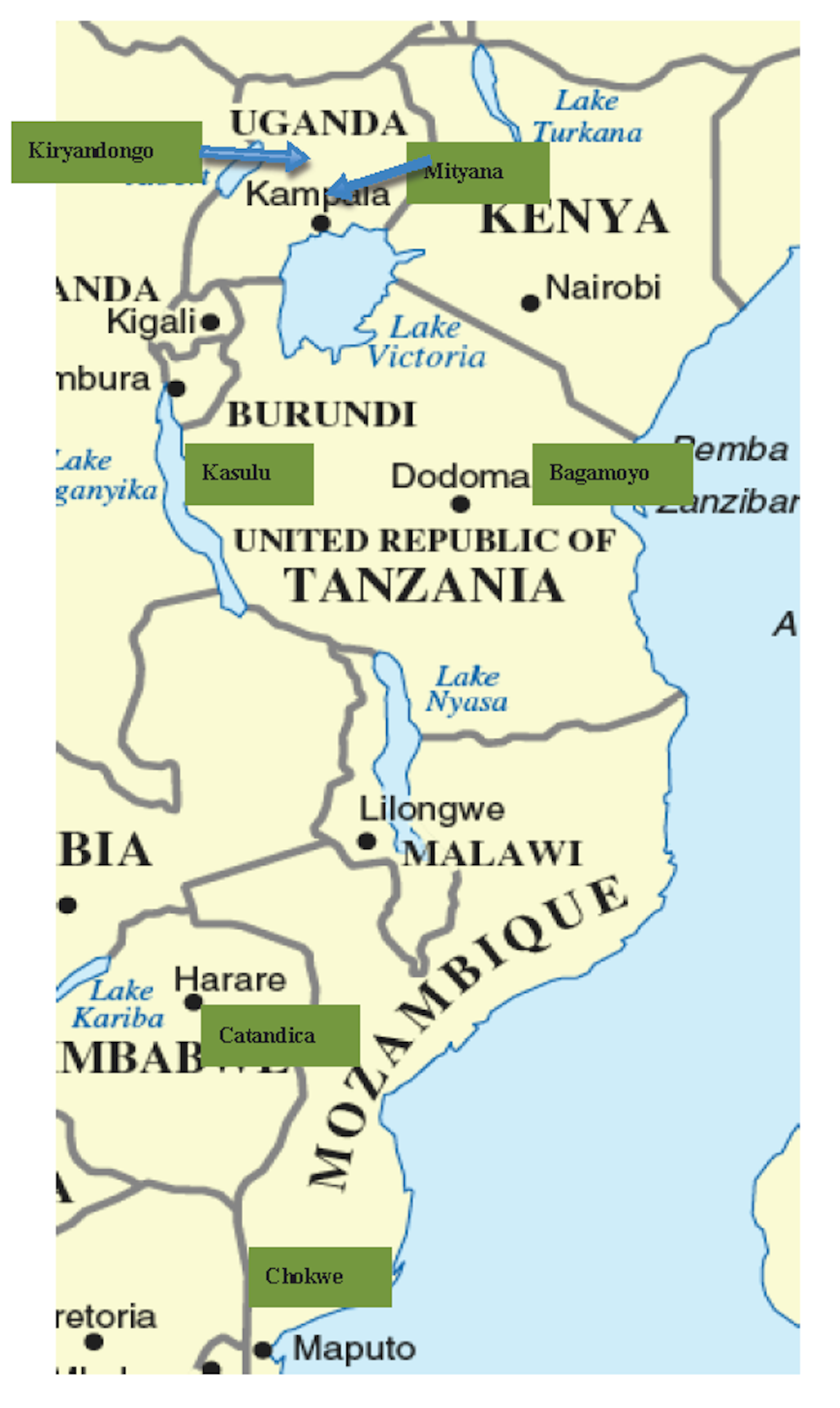

Supplement: Figure S1 — Map of locations of hospitals under investigation. (0.97 MB TIF) [file pmed.1000243.s001.tif]
