# Supplementary material for: Essential Surgery at the District Hospital: A Retrospective Descriptive Analysis in Three African Countries
Source: PLoS Med. 2010 Mar 9;7(3):e1000243. doi: 10.1371/journal.pmed.1000243 (PMC2834708; doi:10.1371/journal.pmed.1000243)
Supplement: Table S1 — Discrepancies between the 3-month individual and 12-month aggregate data. (0.05 MB DOC) [file pmed.1000243.s002.doc]

**Table S1:** Discrepancies between the three-month individual and twelve month aggregate data^1^

|  | Bagamoyo | Kasulu | Chokwe | Catandica | Kiryandongo |
| --- | --- | --- | --- | --- | --- |
| **Percent annual surgeries captured in February, June and October** | | | |  |  |
| Non-obstetric major surgeries | 22.0% | 21.9% | 36.3% | 17.3% | 25.0% |
| Obstetric surgeries | 27.6% | 16.4% | 9.8% | 24.5% | 68.6% |
| Minor surgeries | 3.3% | 0.0% | 1.9% | 23.1% | 24.8% |
|  |  |  |  |  |  |
| **Percent discrepancy**^2^ |  |  |  |  |  |
| ***Major Surgeries*** |  |  |  |  |  |
| Amputation | 0.0% | -1.3% | -1.5% | 3.9% | 1.3% |
| Appendectomy | 4.1% | -1.6% | 4.2% | -2.8% | 0.0% |
| Circumcision | 0.0% | 0.1% | 1.6% | -8.0% | 32.5% |
| Excision | -10.6% | -2.6% | -16.1% | -16.5% | -15.0% |
| Herniorrhaphy | -11.0% | -14.1% | 2.4% | 6.5% | -3.8% |
| Hydrocelectomy | -4.2% | 0.9% | 1.9% | 16.0% | 1.3% |
| Hysterectomy (gynecologic diagnoses) | -1.4% | -7.3% | -0.9% | 0.0% | 1.3% |
| Laparotomy | -3.3% | -23.4% | 8.6% | 1.1% | 0.0% |
| Open fracture reduction | 30.0% | 0.0% | -6.3% | 0.0% | 0.0% |
| Other major surgeries | -6.1% | -26.9% | 11.1% | 5.3% | -17.5% |
|  |  |  |  |  |  |
| ***Obstetric Surgeries*** |  |  |  |  |  |
| Bilateral tubal ligation | 1.2% | -5.7% | 4.7% | 3.6% | 14.3% |
| Caesarian section | 3.0% | -20.3% | 6.9% | 6.1% | 53.5% |
| Evacuation | -3.5% | 22.3% | 0.0% | 0.9% | 0.0% |
| Other obstetric Surgeries | 0.0% | -1.0% | -13.3% | -14.0% | -67.7% |

^1^The individual and aggregate data in Mityana, Buluba and Iganga were both based on 12 months and had few discrepancies, they are not reported in the present table; ^2^Obtained by subtracting the percent distribution from the 12 month aggregate data to the percent distribution from the 3 month individual data: zero means no difference, a negative value means higher rate in the 3 month individual data, a positive value means a lower rate in the 3 month individual data compared to the 12 month aggregate data.
